# Recruitment of Oct4 Protein to UV-Damaged Chromatin in Embryonic Stem Cells

**DOI:** 10.1371/journal.pone.0027281

**Published:** 2011-12-02

**Authors:** Eva Bártová, Gabriela Šustáčková, Lenka Stixová, Stanislav Kozubek, Soňa Legartová, Veronika Foltánková

**Affiliations:** Department of Molecular Cytology and Cytometry, Institute of Biophysics, Academy of Sciences of the Czech Republic, Brno, Czech Republic; St. Georges University of London, United Kingdom

## Abstract

**Background:**

Oct4 is a specific marker of embryonic stem cell (ESC) pluripotency. However, little is known regarding how Oct4 responds to DNA damage. Here, we investigated whether Oct4 recognizes damaged chromatin in mouse ESCs stably expressing GFP-Oct4. These experiments should contribute to the knowledge of how ESC genomic integrity is maintained, which is crucial for potential application of human ESCs in regenerative medicine.

**Methodology/Principal Findings:**

We used time-lapse confocal microscopy, microirradiation by UV laser (355 nm), induction of DNA lesions by specific agents, and GFP technology to study the Oct4 response to DNA damage. We found that Oct4 accumulates in UV-damaged regions immediately after irradiation in an adenosine triphosphate-dependent manner. Intriguingly, this event was not accompanied by pronounced Nanog and c-MYC recruitment to the UV-damaged sites. The accumulation of Oct4 to UV-damaged chromatin occurred simultaneously with H3K9 deacetylation and H2AX phosphorylation (γH2AX). Moreover, we observed an ESC-specific nuclear distribution of γH2AX after interference to cellular processes, including histone acetylation, transcription, and cell metabolism. Inhibition of histone deacetylases mostly prevented pronounced Oct4 accumulation at UV-irradiated chromatin.

**Conclusions/Significance:**

Our studies demonstrate pluripotency-specific events that accompany DNA damage responses. Here, we discuss how ESCs might respond to DNA damage caused by genotoxic injury that might lead to unwanted genomic instability.

## Introduction

Embryonic stem cells (ESCs) display different sensitivities to DNA damage compared with differentiated cells, including mouse embryonic fibroblasts (MEFs) that are used as a feeder layer for ESC cultivation. For example, mouse ESCs (mESCs) are more sensitive to treatment with UV or γ-ray irradiation than differentiated MEFs [1]. These differences can be ascribed to the more open chromatin configuration in ESCs [1], [2]. Such a high sensitivity of ESCs to genotoxic injury increases the probability of nonrepaired DNA lesions, which might lead to genome mutations and subsequent severe malformation in developing organisms [1].

Oct4 is a transcription factor that is critical for the maintenance of the self-renewal and pluripotency of ESCs [3], [4]. *Oct4* is primarily expressed in germline cells, and it could be important for development. Proper *Oct4* transcription is required for the formation of the inner cell mass of blastocysts, and downregulation of *Oct4* is associated with ESC differentiation [3], [5], [6]. *Oct4* collaborates with *Sox* and *Nanog* to maintain ESC pluripotency, and thus, *Oct4*, *Nanog*, and *Sox2* form an interconnected autoregulatory network [7], [8]. From this perspective, it appears likely that Oct4 can modulate the higher order chromatin structure of *Nanog*.

During differentiation, *Oct4* downregulation is accompanied by increased trimethylation of histone H3 at lysine 9 (H3K9me3), which is considered a marker of chromatin repression. Thus, Oct4 is responsible for the decreased binding of RNA Pol II and the insulator protein CTCF at the *Nanog* promoter, which leads to *Nanog* downregulation [9]. *Nanog* transcription is additionally regulated by the Klf4 and Tbx3 proteins [10], [11]. Interestingly, genes that map in close proximity to the *Nanog* locus (within 160 kbp) are regulated by Oct4 [12]. The importance of ESC pluripotency-related genes (*Oct4*, *Sox2*, *c-myc*, and *Klf4*) is also reflected in a relatively new methodological approach for the reprogramming of somatic cells into pluripotent cells. These cells are termed induced pluripotent stem cells (iPSCs), and compared to human ESCs (hESCs), iPSCs have immense therapeutically potential without ethical problems [13].

Pluripotent ESCs are precursors of all cell types in an organism; thus, inappropriate DNA repair can lead to mutations that severely injure the organism during development. A fundamental question concerns the responses of proteins and nuclear substructures to DNA lesions and the accompanying local changes in chromatin conformation [14], [15]. DNA lesions are specifically recognized by several proteins in a hierarchical manner: First, double-strand breaks (DSBs) are recognized by primary protein complexes, such as MRE11-RAD50-NBS1, which is responsible for the activation of a DNA damage-related serine/threonine protein kinase called ataxia telangiectasia mutated (ATM; summarized by [16]). MRE11-RAD50-NBS1 binding and ATM activation are responsible for the phosphorylation of histone H2AX (γH2AX), which is an early event in the repair of DNA lesions [17], [18], [19]. This is accompanied by the binding of the mediator protein MDC1, which recruits chromatin remodeling factors, including 53BP1 and BRCA1. The hierarchical assembly of fundamental proteins involved in DNA repair is accompanied by the recruitment of additional chromatin related factors, such as heterochromatin protein 1 (HP1) or the polycomb group proteins BMI1 and Mel18 [20], [21], [22]. Intriguingly, the depletion of chromatin helicase DNA-binding protein 4 at DSBs disrupts chromatin repair responses at the level of RNF168 ubiquitin ligases, which influence the ubiquitination and assembly of BRCA1 [23].

Based on these observations, we explored the functional properties of Oct4, an important pluripotency factor, in mESCs irradiated by UV laser or exposed to distinct DNA-damaging agents. It is well known that DNA repair mechanisms maintain genomic stability; thus, proper DNA repair is essential, especially during the cultivation of hESCs that are sensitive to genomic instability [24]–[27]. The genomic integrity of hESCs and iPSCs is especially critical given their potential therapeutic applications. Thus, we primarily aimed to determine whether pluripotency-related factors, including Oct4, can be recruited to chromatin-containing DNA lesions in mESCs. These cells were selected because most of the original molecular knowledge of early embryonic development comes from studies of mouse embryos and mESCs [4], [28]. Given that Oct4 has a high degree of sequence similarity among mammalian species (amino acid sequences of human and mouse Oct4 are 87% identical), this factor likely plays a similar role in these species [29], [30].

## Results

### Acetylation-dependent Oct4 recruitment to UV-damaged chromatin in mouse ESCs

**Figure 1:**
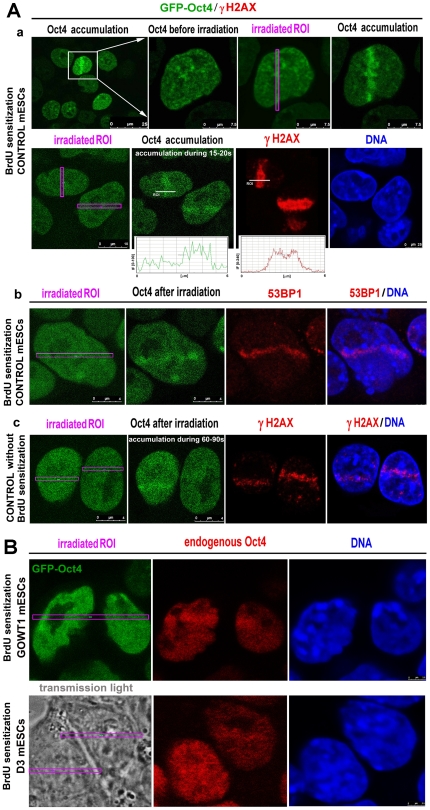
Recruitment of Oct4 to UV-damaged chromatin in GOWT1 mESCs. **A**, GFP-OCT4 was recruited to UV-damaged chromatin 15–20 s after local UV irradiation. Irradiated regions (pink frames) were characterized in **Aa** by increased Oct4 levels (green) and γH2AX positivity (red), which were observed in the cells sensitized by BrdU. **Ab**, Irradiated chromatin with pronounced accumulation of Oct4 was 53BP1-positive (red). **Ac**, Absence of BrdU sensitization was tested. **B**, Endogenous Oct4 (red) was recruited to DNA lesions in both GOWT1 and D3 mESCs, **Scale bars**: in each panel, the scale bar is shown with the relevant value.

In GOWT1 mESCs stably expressing GFP-Oct4, we observed increased Oct4 accumulation at 5-bromo-2′-deoxy-uridine (BrdU)-sensitized and UV laser-irradiated (355 nm) chromatin (Fig. 1Aa). Oct4 accumulation was observed within seconds after irradiation (Fig. 1Aa), and microirradiated genomic regions were γH2AX- and 53BP1-positive (Fig. 1Aa or 1Ab, red). Next, we found slower Oct4 accumulation at UV-induced DNA lesions, and slight γH2AX positivity when BrdU was not used for cell sensitization (Fig. 1Ac). This event could be caused by laser properties or wavelengths [31]. For example, distinct responses of Ku factor and cohesin were also observed upon different experimental conditions leading to the induction of DNA lesions [31].

Although the GFP-Oct4 fusion protein is functional in GOWT1 mESCs [32], we additionally verified that endogenous Oct4 can recognize UV-damaged chromatin (Fig. 1B). In all experiments, we used BrdU sensitization. For irradiation, we detected D3 mESCs under transmission light and GOWT1 cells according to GFP-Oct4 fluorescence. We are aware that, in GOWT1 cells, both endogenous and exogenous Oct4 were detected with the use of a specific antibody; however, in D3 cells, endogenous Oct4 appears to be capable of recognizing UV-induced DNA lesions (Fig. 1B). In these types of experiments, we confirmed the statement of Hong et al. [33] and Suzuki et al. [34] that weak antibody staining of locally irradiated cells can occur owing to low antibody sensitivity or rapid protein dissociation from the regions where the protein accumulates.

**Figure 2:**
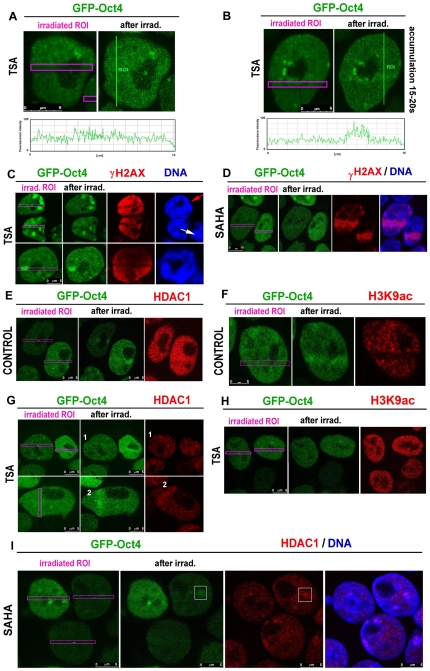
Oct4 recruitment to DNA lesions and acetylation events. **A**, **B**, Panels show the possibilities of how GFP-Oct4 recognizes UV-damaged chromatin after treatment of cells with the HDACi TSA. **C**, Oct4 and γH2AX levels in UV-irradiated ROIs of TSA-treated cells. **D**, Oct4 and γH2AX levels in UV-irradiated ROIs of SAHA-treated cells. **E**, Oct4 and HDAC1 levels in UV-irradiated ROIs of nontreated control cells. **F**, Oct4 and H3K9 acetylation levels in UV-irradiated ROIs of nontreated control cells. **G**, Oct4 and HDAC1 levels in UV-irradiated ROIs of TSA-treated cells. **H**, Oct4 and H3K9 acetylation levels in UV-irradiated ROIs of TSA-treated cells. **I**, Oct4 and HDAC1 levels in UV-irradiated ROIs of SAHA-treated cells. **Scale bars**: in each panel, the scale bar is shown with the relevant value.

Here, we also tested how changes in histone acetylation can influence Oct4 recruitment to UV-induced DNA lesions. The accumulation of GFP-Oct4 at DNA lesions was affected by an inhibitor of histone deacetylases (HDACi), trichostatin A (TSA), and a clinically promising HDACi, SAHA (vorinostat) (Fig. 2). In TSA-treated GOWT1 mESCs, Oct4 accumulation at irradiated regions was lower than that detected in nontreated cells (compare Fig. 1Aa with 2A and B or Table 1). TSA additionally increased global cellular γH2AX positivity, but this was not observed at UV-irradiated chromatin that was absent of this specific histone marker (Fig. 2C). Conversely, SAHA treatment caused pronounced γH2AX accumulation at only UV-irradiated regions, and Oct4 only subtly recognized DNA lesions (Fig. 2D). In control cells, we observed the rule, when Oct4 expression was increased after UV irradiation, the expression of HDAC1 as also increased (Fig. 2E). This event was accompanied by decreased H3K9 acetylation (Fig. 2F). When the cells were treated by TSA, the HDAC1 level was low in both non-irradiated and irradiated regions (Fig. 2G, cell 1). However, in the cells with Oct4 accumulation at UV-damaged chromatin, pronounced HDAC1 accumulation was observed (Fig. 2G, cell 2 and Table 1). In TSA-treated cells with no Oct4 accumulation at UV-damaged regions, we observed high levels of H3K9 acetylation (Fig. 2H). The effect of SAHA was similar to that of TSA treatment (see Table 1), but we additionally noticed that after SAHA treatment, Oct4 foci were HDAC1-positive (frame in Fig. 2I, frame).

**Table 1:** Recruitment of selected proteins to UV-damaged chromatin.

protein	treatment	number of cells	accumulation	stable level	decreased level	whole cell positivity	note
Oct4	control	116	101	15	0	0	Strong accumulation of Oct4
	TSA	118	28	98	0	0	Slight accumulation of Oct4
	SAHA	107	33	74	0	0	Slight accumulation of Oct4
	Actinomycin D	101	0	28	73	0	No accumulation of Oct4
	ATP depletion	74	0	6	68	0	100% decondensation of chromatin (in DAPI), different pattern of cell nuclei (more foci in DAPI and Oct4 positive foci). No accumulation of Oct4.
	without BrdU	102	17	85	0	0	Slight and later accumulation of Oct4 compared to BrdU sensitization
C-myc	control	51	0	51	0	0	Stable level of C-myc
Nanog	control	61	0	61	0	0	Stable level of Nanog
HDAC1	control	35	14	21	0	0	High level of HDAC1
	TSA	32	8	24	0	0	Accumulation of HDAC1 when accumulation of Oct4 occurs
	SAHA	41	12	29	0	0	Foci of Oct4 were HDAC1 positive–
H3K9Ac	control	30	0	10	20	0	No H3K9ac in UV-irradiated chromatin
	TSA	28	0	28	0	0	Increased level of H3K9ac in comparison to control
	Actinomycin D	25	0	10	15	0	Low level of H3K9ac in UV-irradiated chromatin
	ATP depletion	18	0	0	18	0	Decreased H3K9ac as a result of 100% decondensation of chromatin (in DAPI), different pattern in cell nuclei (more foci in DAPI and Oct4 positive foci)
γH2AX	control	105	90	0	0	15	Pronounced accumulation of γH2AX
	TSA	24	0	0	6	18	Decreased level of γH2AX appeared in UV irradiated regions but entire genome was γH2AX positive
	SAHA	41	36	0	0	5	γH2AX positivity in UV-irradiated regions
	Actinomycin D	29	10	0	0	19	High level of γH2AX in entire genome
	ATP depletion	35	0	35	0	0	100% decondensation of chromatin (in DAPI), different pattern in cell nuclei. Low γH2AX positivity.
	without BrdU	65	61	0	0	4	Low level of γH2AX positivity in UV-irradiated regions

### Adenosine triphosphate (ATP)-dependent recruitment of Oct4 to UV-damaged chromatin is not accompanied by pronounced Nanog and c-MYC accumulation

**Figure 3:**
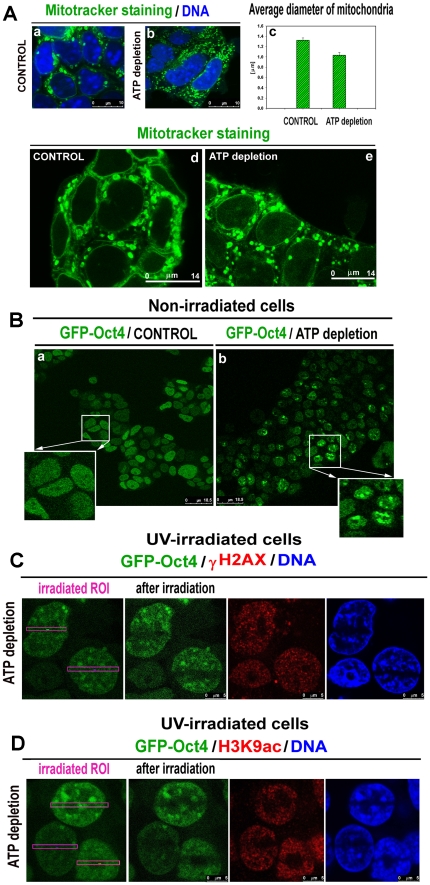
ATP depletion and Oct4 levels at UV-damaged chromatin. **A**, In comparison with control (**a**), ATP depletion reduced the diameter of mitochondria (**b**). **c**, Panel shows the comparison of the average diameter of mitochondria in control cells and ATP-depleted cells. **d**, Mitochondria staining in control cells. **e**, Mitochondria staining after ATP depletion. **B**, In comparison with control nonirradiated cells (**a**), GFP-Oct4 become focally distributed after ATP depletion (**b**). **C**, DNA lesions were induced in GOWT1 mESCs after ATP depletion, but the absence of GFP-Oct4 accumulation was observed. γH2AX positivity was low and homogeneous within entire cells. **D**, H3K9 acetylation levels were low in UV-irradiated chromatin after ATP depletion. **Scale bars**: in each panel, the scale bar is shown with the relevant value.

Previous studies demonstrated that the movement of some nuclear proteins is energy-dependent. Kruhlak et al. [35] demonstrated ATP-dependent chromatin remodeling and the recruitment of DNA damage repair factors to irradiated chromatin. Thus, we performed ATP depletion experiments following the protocol of Kruhlak et al. [35]. Changes in mitochondrial morphology after induced ATP depletion were assayed using Mitotracker (Fig. 3A). In comparison with nontreated control cells (Fig. 3Aa and Ad), ATP depletion reduced the diameter of mitochondria (see Fig. 3Ab, Ae and quantification in Fig. 3Ac). Interestingly, ATP depletion by itself (without irradiation) caused Oct4 accumulation into foci (compare nonirradiated control cells in Fig. 3Ba with Fig. 3Bb). Following ATP depletion, we did not observe pronounced accumulation of Oct4 at UV-irradiated chromatin, which was slightly γH2AX-positive (Fig. 3C). With these experiments, we confirmed that ESCs require ATP for the proper recruitment of specific proteins to chromatin with DNA lesions. Thus, optimal mitochondrial function is critical. Additional to these experiments, we examined whether ATP depletion induced changes in H3K9 acetylation within UV-irradiated regions. We observed decreased H3K9 acetylation at DNA lesions, which was additionally accompanied by reduced Oct4 levels and chromatin decompaction after ATP depletion and UV irradiation (compare control cells in Fig. 1A with Fig. 3C–D, 4′,6-diamidino-2-phenylindole [DAPI] staining shown in blue).

**Figure 4:**
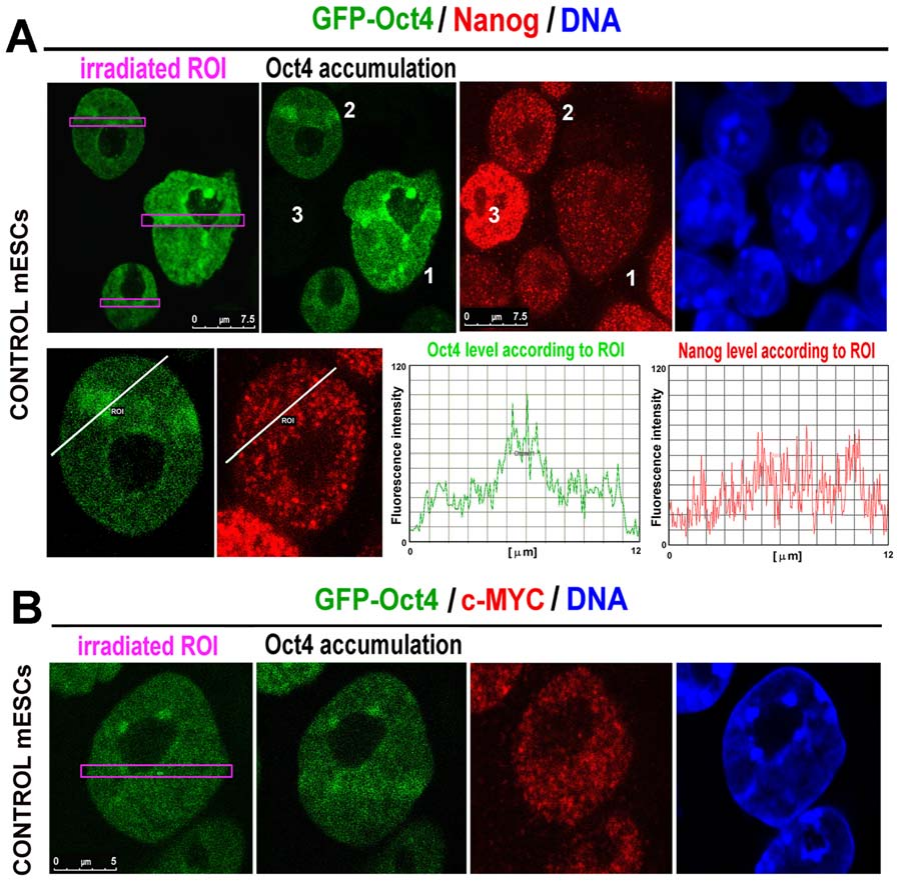
Detection of Nanog and c-MYC at UV-irradiated regions. The cells with accumulated GFP-Oct4 (green) had relatively stable levels of Nanog (red, **A**) and c-MYC protein (red, **B**) at UV-irradiated chromatin. Quantification of the protein levels in the graphs of panel A was performed using LEICA LAS AF software (version 2.1.2.). **Scale bars**: in each panel, the scale bar is shown with the relevant value.

Next, we addressed whether pronounced Oct4 recruitment to UV-damaged chromatin occurs in parallel with the activation of other pluripotency-related proteins, such as Nanog and c-MYC. In this case, we did not observe pronounced accumulation of either Nanog or c-MYC at UV-irradiated regions with significant Oct4 accumulation (Fig. 4A and B). Because these proteins were not completely absent from irradiated regions, we cannot rule out that certain levels of Nanog or c-MYC are sufficient for other protein binding at irradiated chromatin. Interestingly, cells with globally lower levels of Nanog were characterized by pronounced Oct4 recruitment to UV-damaged chromatin (Fig. 4A, compare cell 1 with lower Nanog levels and cell 2 with higher Nanog levels and see Oct4 levels. Also note cell 3, which lacks Oct4 and has a very high level of Nanog).

### Recruitment of Oct4 to UV-damaged chromatin and phosphorylation of H2AX

**Figure 5:**
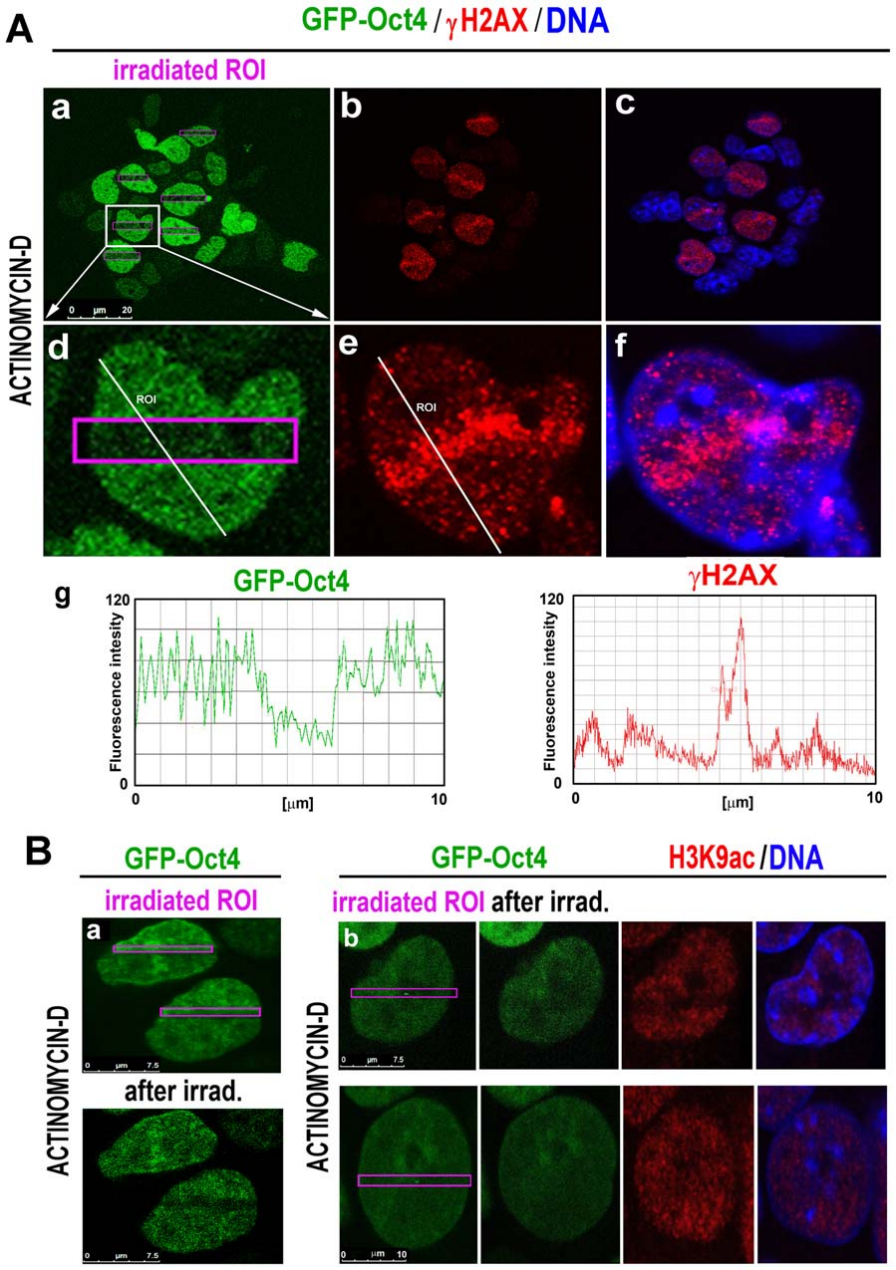
GFP-Oct4 at UV-damaged chromatin and inhibition of transcription. **A**, GFP-Oct4 levels in GOWT1 mESCs after the inhibition of transcription by actinomycin D treatment are shown. Treatment with actinomycin D reduced the level of Oct4 (panels **a** and **d**) in regions of laser-generated DNA lesions, which were γH2AX-positive (red) (panels **b** and **e** show γH2AX; panels **c** and **f** show the overlay of γH2AX with all DNA content). Protein levels of Oct4 (green) and γH2AX (red) were quantified using LEICA LAS AF (version 2.1.2.) software, and the results are shown in panel **g**. **Ba**, Panels show the possibilities of GFP-Oct4 (green) levels in UV-induced DNA lesions after actinomycin D treatment. **Bb**, H3K9 acetylation (red) at irradiated chromatin of actinomycin D-treated cells was slightly reduced or not changed in comparison with that in nonirradiated areas of the same cell.

The morphology of γH2AX-positive regions after UV irradiation indicates a fast rearrangement of locally irradiated chromatin in GOWT1 mESCs, which proliferate at a much faster rate than somatic cells. In many cases, we observed fast movement of cells, cell nuclei, and entire mESC colonies over several minutes. This fast movement also influenced the shape of γH2AX-positive regions compared to that of the originally irradiated regions of interest (ROIs; Fig. 5A).

In nontreated control cells, we observed γH2AX-positive regions after irradiation as expected. However, in many cases, the γH2AX-positive area was wider than the irradiated area (Fig. 1Aa, red). After TSA treatment, but not after SAHA treatment, we noticed that the irradiated region was absent of γH2AX, but entire nuclei were highly positive for γH2AX compared to nuclei of control cells (compare Figs. 1Aa and 2C). This observation is in agreement with that of Bakkenist and Kastan [36], who reported that ATM activation occurs after TSA treatment.

Here, we also examined the effect of other chromatin-modifying events on the nuclear distribution of γH2AX. For example, ATP depletion was associated with slight γH2AX positivity across the entire nuclear volume (Fig. 3C, red). However, inhibition of transcription by actinomycin D induced strong γH2AX accumulation at irradiated chromatin and in entire nuclei (Fig. 5Aa–f, quantification of protein levels across the selected ROI is in Fig. 5Ag).

### Oct4 level in UV-irradiated chromatin and inhibition of transcription

When we assayed the recruitment of Oct4 to DNA lesions, we observed that inhibition of transcription (disturbed transcription elongation) via actinomycin D treatment significantly affected Oct4 recruitment to UV-damaged chromatin (Fig. 5A and B, Table 1). In this case, we investigated how Oct4, as a transcription factor, responds to the inhibition of transcription. In many cases, actinomycin D-treated mESCs displayed significantly reduced levels of Oct4 and H3K9 acetylation at irradiated chromatin (Fig. 5A, 5Ba and upper panels in Fig. 5Bb). However, in some cases, relatively stable levels of Oct4 were accompanied by stable H3K9 acetylation level (lower panels in Fig. 5Bb).

### Spontaneously occurring DNA damage and induced DNA damage-associated foci

**Figure 6:**
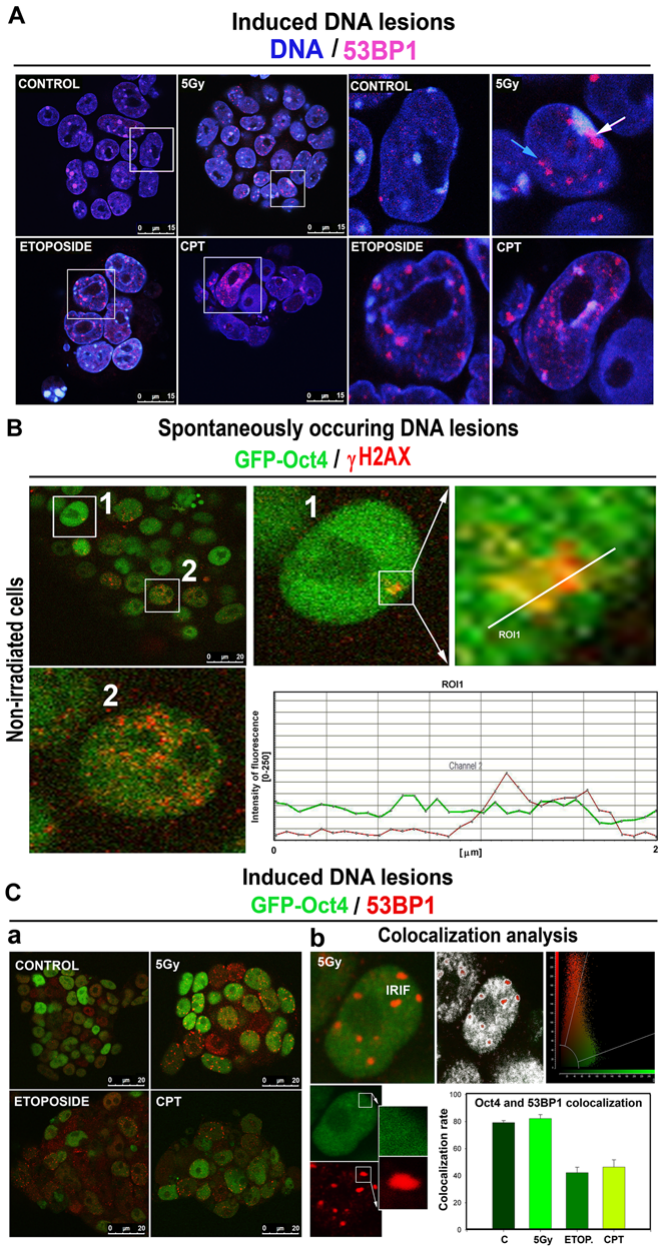
Accumulation of γH2AX and appearance of 53PB1-positive regions in spontaneously occurring or induced DNA lesions. **A**, Appearance of 53BP1-positive foci (red) in heterochromatin (accumulated blue) and euchromatin (dispersed blue) nuclear regions of control, γ-irradiated, and CPT or ETOP-treated cells. **B**, Spontaneously occurring γH2AX foci (red) that overlap with GFP-Oct4-positive regions (green) in GOWT1 mESC nuclei. Quantification in the graph was performed using LEICA LAS AF software (version 2.1.2.). **Ca**, GFP-Oct4 and 53BP1 nuclear patterns in control nonirradiated cells, **γ**-irradiated cells, and CPT or ETOP-treated cells. **Cb**, As an example, 53BP1-positive IRIF (red) and analysis of Oct4 and 53BP1 colocalization are shown. The level of colocalization was evaluated using a quantification tool from LEICA LAS AF software (version 2.1.2.), and the results are shown in the bar chart as mean±S.E. **Scale bars**: in each panel, the scale bar is shown with the relevant value.

We also analyzed the appearance and nuclear distribution of spontaneously occurring DNA lesions, γ-radiation-induced foci (ionizing radiation-induced foci, IRIF), or camptothecin (CPT) and etoposide (ETOP)-stimulated 53BP1-positive regions (Fig. 6A). In mESCs, we observed the appearance of 53BP1-positive signals in clusters of both heterochromatin (chromocenters, 6A white arrow) and euchromatin regions (Fig. 6A, blue arrow).

Additionally, we examined the appearance of Oct4 in spontaneously occurring γH2AX-positive foci in nonirradiated cells (Fig. 6B). In mESCs, GFP-Oct4 is homogeneously dispersed throughout the interphase nuclei; thus, Oct4 colocalizes with spontaneously occurring γH2AX-positive or 53BP1-positive foci (Fig. 6B or C bar chart). In this case, we did not observe focal accumulation of GFP-Oct4 in γH2AX- or 53BP1-positive chromatin regions (Fig. 6B and C). Despite the relatively stable levels of Oct4, which homogeneously appears throughout the mESC nucleus, we can neither confirm nor exclude the role of Oct4 in recognizing spontaneously occurring DNA lesions or IRIF, CPT- and etoposide-induced DNA lesions (Fig. 6B and Ca). Moreover, using a Leica software colocalization tool, we detected a relatively high degree of colocalization between Oct4 and 53BP1-positive regions (Fig. 6Cb, bar chart).

### OCT4 fluorescence recovery after photobleaching in nonirradiated and irradiated regions

**Figure 7:**
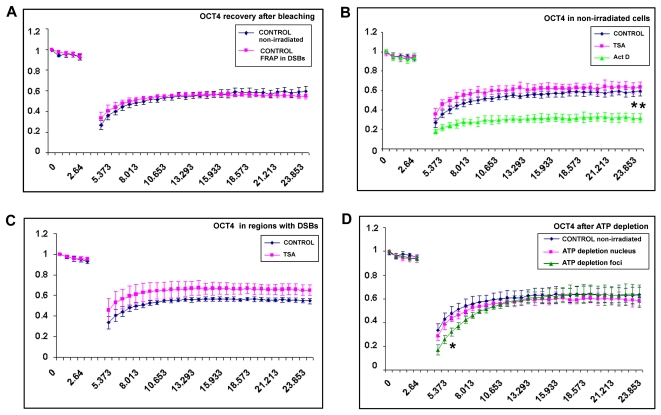
Oct4 kinetics in irradiated and nonirradiated regions. **A**, FRAP was used to study Oct4 kinetics in nonirradiated mESCs and GFP-Oct4 recovery in UV-damaged chromatin. **B**, GFP-Oct4 recovery after photobleaching was studied in control nonirradiated cell nuclei (blue line) and compared with that in TSA-treated (magenta line) and actinomycin D-treated (green line) cells. **C**, FRAP was performed in UV-irradiated regions with pronounced Oct4 accumulation (control, blue line) and with unchanged levels of Oct4 after TSA treatment (magenta line). **D**, FRAP data for Oct4 in nonirradiated cells undergoing ATP depletion compared to that in nontreated control cells (blue line). After ATP depletion, Oct4 recovery was measured in the nucleoplasm when Oct4 did not accumulate into foci (magenta line) and when GFP-Oct4 was accumulated into foci (green line). One (two) asterisk(s) show(s) statistically significant differences at p≤0.05 (p≤0.01).

We observed that Oct4 was recruited to UV-damaged chromatin immediately after local microirradiation. This feature of Oct4 enabled us to perform fluorescence recovery after photobleaching (FRAP) at laser-injured chromatin. In nonirradiated control cells, we observed fast GFP-Oct4 recovery similarly as observed in chromatin with DNA lesions (Fig. 7A). When the cells were treated by selected agents, fluorescence recovery 6 s after photobleaching (*R_6_*) was 51% in control cells and 60% in TSA-treated cells. In nonirradiated cells, suppression of transcription by actinomycin D was associated with a slow recovery of GFP-Oct4; (*R_6_*) was 29% (asterisks in Fig. 7B show statistically significant results at p≤0.01). When we performed FRAP on UV-irradiated chromatin, we detected significantly increased GFP-Oct4 recovery after TSA treatment; (*R_6_*) was 65%, but in control cells, (*R_6_*) was 55% (Fig. 7C). After actinomycin D treatment, it was not possible to measure FRAP in UV-irradiated regions because in the majority of irradiated cells, there was an absence of GFP-Oct4 (Fig. 5Ba, green nuclei). When we performed FRAP after ATP depletion in an area of the nucleus without Oct4 foci, we found no significant changes in Oct4 recovery compared to control values obtained in nonirradiated cells (Fig. 7D). However, when Oct4 was accumulated into foci (after ATP depletion, Fig. 3Bb), we observed significantly slower recovery after photobleaching (asterisk in Fig. 7D shows statistically significant results at p≤0.05).

## Discussion

With the increased importance of fluorescence tag protein technologies and advanced approaches for live cell confocal microscopy, it is possible to more precisely study the functional aspects of particular chromatin domains and nuclear substructures. It is now possible to reveal correlations between function and nuclear structure for the protein complexes that recognize DNA lesions. ESCs represent a unique system to study the DNA repair hierarchical machinery, as these are the cells with the highest level of chromatin decondensation [1], [2], [37] and specific pluripotency-related protein levels [3], [5], [6]. ESCs are also interesting because proper recognition of DNA lesions and their repair is crucial for normal embryonic development. It is well known that initial studies in mESCs contributed to the first isolation and cultivation of human ESCs [4], [28]. Furthermore, human iPSCs have immense potential for the study of pluripotency in human cells. In terms of DNA damage responses (DDRs), hESCs and human iPSCs are highly similar, with both exhibiting hypersensitivity to DNA damaging agents compared to somatic cells [38]. In a study of mouse ESCs, de Waard et al. [1] described the so-called double safeguard mechanism against mutations. This event can prevent undesirable embryonic malformation during prenatal development. Thus, general knowledge of how ESCs protect themselves against oxidative stress and other genotoxic injuries, inducing DNA lesions, is important for understanding of how genomic integrity is maintained.

Here, we have concluded that Oct4, an important pluripotency-related transcription factor, is recruited to chromatin-containing DNA lesions in mESCs. Rapid and significant recruitment of GFP-Oct4 to UV-irradiated chromatin was abrogated by HDAC inhibition and transcriptional suppression (compare Fig. 1Aa with Figs. 2A–B and 5A–B). Interestingly, the nuclear distribution of γH2AX, a prominent marker of DNA injury, did not correspond to Oct4 protein levels in the UV-damaged chromatin of TSA-treated cells (Fig. 2C). However, the increased level of γH2AX in entire cells after TSA-stimulation is in agreement with the finding of Bakkenist and Kastan [36] that ATM activation occurs after TSA treatment. Taken together, our data confirm that histone post-translational modifications, including phosphorylation and acetylation, play important roles in DDRs (summarized by [39]).

More than 20 years ago, it was discovered that the hyperacetylation of histones is an important event following UV irradiation. It was demonstrated that DNA repair is more efficient in hyperacetylated nucleosomes [40], [41]. These data have led some researchers to conclude that acetylation makes DNA more accessible to proteins that recognize DNA lesions. However, we found rather hypoacetylation of H3K9 at DNA lesions (Fig. 2F). According to our results, local chromatin decondensation at DSBs, as described by Kruhlak et al. [42], can occur (Fig. 3C–D, blue), but it is not likely that this “open chromatin conformation” is associated with histone hyperacetylation. This finding supports the fact that heterochromatin-related proteins with no affinity to histone acetylation, including HP1, BMI1, and Mel18, recognize DNA lesions [20]–[22], [43]. Thus, possible chromatin decompaction after UV irradiation appears to be another type of chromatin relaxation caused by DNA breaks that is associated with a specific histone signature. Here, we have confirmed that protein recruitment to UV-damaged chromatin is dependent on the histone acetylation state, but hyperacetylation prevents the recruitment of some proteins to DNA lesions (Fig. 2A, B and Table 1). As evidence, histone hyperacetylation also abrogated the recruitment of the polycomb group-related (PcG) protein BMI1 to UV-damaged chromatin [43], and this event is likely influenced by polyadenosine diphosphate ribose polymerase activity (PARP), as reported by Chou et al. [22].

We further observed that inhibition of transcription elongation by actinomycin D significantly abrogated the recruitment of GFP-Oct4 to chromatin with DSBs (Fig. 5). Potential transcriptional inactivity in DSBs indicates the recruitment of heterochromatin-related proteins such HP1 or PcG proteins [20]–[22], [43]. However, we recently noticed that actinomycin D also abrogated the recruitment of these proteins to UV laser-irradiated chromatin [43]. Thus, it is a contradiction, similarly as the recognition of DNA lesions by the transcription factor Oct4. These observations indicate the specific function of the aforementioned proteins at chromatin with DNA lesions. Thus, further analyses of how transcription proceeds in close proximity to DSBs are demanded. Such new information could be valuable for understanding how genomic integrity is controlled, even when 1×10^4^–1×10^5^ genomic lesions must be repaired daily [2].

**Figure 8:**
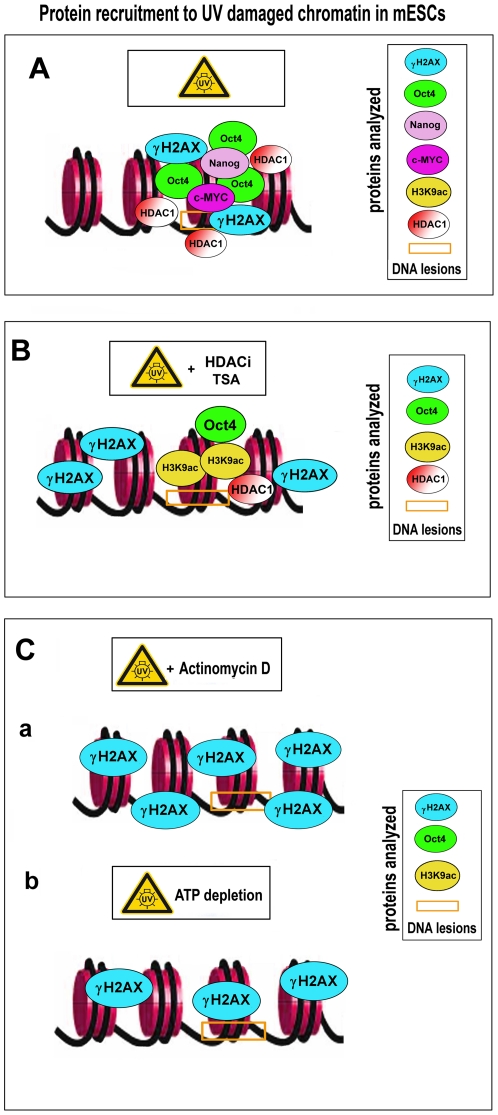
Model of Oct4 accumulation at UV-damaged chromatin. **A**, Oct4 was significantly accumulated at chromatin with laser-induced DNA lesions. In these regions, the levels of Nanog and c-MYC were not changed. This event was accompanied by γH2AX accumulation at DNA lesions. **B**, TSA-induced changes in acetylation stopped the increased accumulation of Oct4 at UV-damaged chromatin. Phosphorylation of H2AX was not observed in the irradiated regions of TSA-treated cells, but high levels of γH2AX were found in nonirradiated regions of these cells. **Ca**, High levels of γH2AX were observed after actinomycin D treatment in both irradiated chromatin and the entire genome. These levels were accompanied by an absence of Oct4 at UV-irradiated chromatin, and there were subtle levels of H3K9 acetylation. **Cb**, ATP depletion did not induce pronounced Oct4 accumulation at irradiated chromatin, and in many cases, there was an absence of Oct4 in UV-damaged genomic regions. After ATP depletion, the H3K9 acetylation level was very low, and γH2AX slightly appeared throughout the entire genome.

In summary, the recruitment of bona fide DNA repair proteins to DNA lesions involves additional events, including the action of heterochromatin-related proteins or ATP-dependent chromatin remodelers that are potentially responsible for DNA repair. ATP-dependent chromatin changes at DSBs have been reported by Kruhlak et al. [35], who demonstrated the restricted movement of DNA lesions and the importance of ATP for the initial detection of injured chromatin. Moreover, this event proceeds independently of γH2AX. Similarly, chromatin decompaction at sites of DNA lesions occur independently of γH2AX [35]. Here, we have demonstrated that the nuclear redistribution and appearance of γH2AX after irradiation is highly specific in mESCs, especially after interference to cellular processes, including histone acetylation, transcription, and cell metabolism (summary in Fig. 8A–C). In addition, phosphorylation of H2AX seems to be unique in mESCs, as shown by Banáth et al. [44] who found that γH2AX-positive foci can appear in the absence of measurable DSBs. Interestingly, differentiation of mESCs is accompanied by reduced histone acetylation, decreased γH2AX foci intensity, and changes in chromatin plasticity, including dynamic exchange of some histones [44], [45], [46]. The notion that Oct4 has an original function in ESCs is strengthened by the fact that Oct4 has the ability to recognize UV-damaged chromatin (Fig. 8A). In addition to ESCs, the *Oct4* gene was found to be expressed in human epithelial dysplasia, which can be associated with tumor growth [47]. Thus, knowledge of the role of Oct4 in DDRs is of functional significance even for anticancer approaches, including radiotherapy.

## Materials and Methods

### ESC cultivation, fixation, and induction of DNA lesions

GOWT1 mESCs stably expressing exogenous Oct4 (a generous gift from Hitoshi Niwa, Laboratory for Pluripotent Stem Cell Studies, RIKEN Center for Developmental Biology, Japan) and D3 mouse embryonic stem cells were cultivated in mESC medium (D-MEM, Dulbecco's Modified Eagle Medium, PAN Biotech, GmbH, Germany) containing 4.5 g/l glucose, 15% fetal calf serum (tested for ESCs), 1× nonessential amino acids (Invitrogen, CZ), 10,000 IU/ml penicillin, 10,000 µg/ml streptomycin, 29.2 mg/ml l-glutamine, and leukemia inhibitory factor (LIF, final concentration 5 ng/ml, Chemicon International, #LIF1010). Cells were cultivated at 37°C in a humidified atmosphere containing 5% CO_2_. GOWT1 cells were treated at 70% confluence with 100 nM TSA for 4 h (Sigma-Aldrich, CZ, # T8552), 8 µM SAHA for 4–6 h (Cayman Chemical, USA, #10009929), or 0.5 µg/ml Actinomycin D for 2 h (#A9415, Sigma-Aldrich, CZ). Then, individual cells were microirradiated using a UV laser (355 nm), which took 1–2 h. During this interval, we irradiated more than 25 nuclei for each experimental event. Experiments were repeated 2–3 times. When we finished the UV irradiation of nuclei, the cells were washed once by PBS and fixed for 20 min by 4% paraformaldehyde. The cell number analyzed is mentioned in Table 1 (some irradiated cells flaked off during immunostaining).

For studies of the recruitment of endogenous Oct4 to UV-irradiated chromatin, D3 mESCs were also sensitized with BrdU and for micro-irradiated approaches, these cells were recognized under transmission light. Endogenous Oct4 was visualized by immunostaining using anti-Oct3/4 (Santa Cruz Biotechnology, USA, #sc-5279).

To induce DNA damage, we additionally used γ-irradiation by Co-60 [48] or cell treatment by DNA-damaging agents, such as campthothecin (CPT, 10 µM, Sigma, CZ, #C9911) or etoposide (ETOP, 10 µM, Sigma, CZ, #E1383). Cells were treated by CPT or ETOP for 24 h. Four hours after γ-irradiation, the cells were fixed for immunostaining by anti-53BP1 (Abcam, UK, #ab21083).

### BrdU sensitization prior to UV irradiation and verification of induced DNA lesions

GFP-Oct4-GOWT1 cells were cultivated under standard conditions. Twenty-four hours after passaging, colonies of GOWT1 mESCs were sensitized with 10 µM BrdU. All experiments, excluding that for data shown in Fig. 1Ac and 1B, were performed with BrdU sensitization. BrdU was added to the cells 16–18 h before microirradiation. For experiments, cells were placed in the cultivation hood (EMBL Heidelberg, Germany) maintained at 37°C and 5% CO_2_. BrdU-sensitized GOWT1 cells were irradiated by a UV laser (355 nm) connected to a Leica TSC SP-5X confocal microscope. The defined ROI was irradiated by 80% laser output that was not reduced using an acusto-optic tunable filter. The following settings were used: 512×512 pixels, 400 Hz, bidirectional mode, 64 lines, zoom >×5–10. Microirradiated cells were fixed in 4% formaldehyde, and γH2AX and 53BP1, markers of UV-damaged chromatin, were detected using a rabbit polyclonal antibodies against γH2AX (phospho S139; Abcam, #ab2893) and 53BP1 (Abcam, #ab21083), respectively.

For immunostaining, the cells were fixed as soon as possible after irradiation. We irradiated only 8–10 cells in one tissue culture dish, and we used 3–4 dishes with irradiated cells. Cells were irradiated for 15–20 min and then fixed immediately. Cells were fixed in 4% paraformaldehyde for 15 min. These experiments showed that Oct4 likely recognizes DNA lesions immediately after irradiation, but disappears after several minutes.

### Immunostaining of interphase nuclei

After inducing DNA lesions, cells were shortly washed once using PBS, and then the cells were fixed for 20 min in 4% formaldehyde and permeabilized with 0.1% Triton X100 for 8 min and with 0.1% saponin (Sigma, Germany) for 12 min. The cells were then washed twice in phosphate buffered saline (PBS) for 15 min. Cells were incubated for 1 h at RT in 1% bovine serum albumin dissolved in PBS. The slides were washed for 15 min in PBS, and preparations were incubated overnight with anti-γH2AX (phospho S139; Abcam, #ab2893), anti-53BP1 (Abcam, #ab21083), anti-c-MYC (N-262) (Santa Cruz Biotechnology, #sc-764), Nanog (Abcam, #ab21603), anti-HDAC1 (Sigma, Germany, #HPA029693), and anti-acetyl H3K9 (Upstate-Millipore, USA, #06-942). The cells were then washed twice in PBS for 5 min and incubated for 1 h with the appropriate secondary antibody: anti-rabbit IgG-FITC (#F0511, Sigma, Germany) or goat anti-mouse IgG3-Alexa Fluor 594 (Molecular Probes, USA). Similar to primary antibodies, the secondary antibodies were diluted 1∶200 in 1% bovine serum albumin dissolved in PBS. Immunostained preparations were washed three times in PBS for 5 min and DAPI was used as a counterstain. After microirradiation (the first step), the immunostained cells (the second step) were observed on cultivation dishes (50-mm Glass Bottom Dishes, No. 1, MatTek Corporation, USA, #P50G-0-30-F) according to the indicated grids. Image acquisition was performed by the conventional mode using a Leica TCS SP-5X confocal microscope. To scan immunostained nuclei, we used a white light laser (WLL, 470–670 nm in 1-nm increments), objective magnification was ×64, numerical aperture (N.A.) = 1.4. We additionally used bidirectional X scanning mode, format 1024×1024 pixels, speed 400 Hz, line average 8, frame average 1 and pixel size 240.5 nm×240.5 nm. The number of cells with or without pronounced recruitment of protein of interest was counted manually and independently by two lab members (see results in Table 1). The data in Table 1 are shown in absolute numbers.

### FRAP

FRAP was performed on a Leica TSC SP-5X confocal microscope. For FRAP, we used an argon laser (488 nm) and ×64 magnification/N.A. = 1.4. The cells were cultivated on 50-mm Glass Bottom Dishes (No. 1, MatTek Corporation, USA, #P50G-0-30-F) in a cultivation hood (EMBL Heidelberg, Germany) equipped with an “Air Stream” incubator and heated to 37°C. The cells were maintained at 5% CO_2_ for optimal cell growth. FRAP was performed following Bártová et al. [49] and Stixová et al. [50]. Statistical analysis was performed using Student's t-test.

### ATP depletion

Cells were incubated in PBS containing 10% fetal bovine serum, 10 mM 2-deoxyglucose, 10 mM sodium azide, and 7.5 mg/ml Hoechst dye. We used the protocol of Kruhlak et al. [35]. ATP depletion was verified by mitochondrial morphology assayed using Mitotracker (Invitrogen, CZ) staining.
